# Microencapsulation by Membrane Emulsification of Biophenols Recovered from Olive Mill Wastewaters

**DOI:** 10.3390/membranes6020025

**Published:** 2016-05-09

**Authors:** Emma Piacentini, Teresa Poerio, Fabio Bazzarelli, Lidietta Giorno

**Affiliations:** Institute on Membrane Technology, National Research Council, ITM-CNR, Via P. Bucci 17/C at University of Calabria, Calabria 87036, Italy; t.poerio@itm.cnr.it (T.P.); f.bazzarelli@itm.cnr.it (F.B.); l.giorno@itm.cnr.it (L.G.)

**Keywords:** membrane emulsification, water-in-oil emulsions, biophenols, olive mill wastewater, encapsulation, drug delivery

## Abstract

Biophenols are highly prized for their free radical scavenging and antioxidant activities. Olive mill wastewaters (OMWWs) are rich in biophenols. For this reason, there is a growing interest in the recovery and valorization of these compounds. Applications for the encapsulation have increased in the food industry as well as the pharmaceutical and cosmetic fields, among others. Advancements in micro-fabrication methods are needed to design new functional particles with target properties in terms of size, size distribution, and functional activity. This paper describes the use of the membrane emulsification method for the fine-tuning of microparticle production with biofunctional activity. In particular, in this pioneering work, membrane emulsification has been used as an advanced method for biophenols encapsulation. Catechol has been used as a biophenol model, while a biophenols mixture recovered from OMWWs were used as a real matrix. Water-in-oil emulsions with droplet sizes approximately 2.3 times the membrane pore diameter, a distribution span of 0.33, and high encapsulation efficiency (98% ± 1% and 92% ± 3%, for catechol and biophenols, respectively) were produced. The release of biophenols was also investigated.

## 1. Introduction

In the last several years, olive mill wastewaters (OMWWs) have received increasing attention because of their high content of biophenols, which possess a high spectrum of biological functions, which include antioxidant, anti-inflammatory, antibacterial, and antiviral activities [1,2,3]. The high polar nature of most olive oil biophenolic compounds result in their sizable loss with the wastewater during processing [4]. Several approaches have been investigated for biophenol recovery for their potential reuse in functional foods, nutraceutical, and pharmaceutical products. Navarro *et al.* [5] used ultrafiltration and nanofiltration of OMWWs and successive spray drying with maltodextrin and acacia fiber as antiglycative ingredients for foods and pharmacological preparations. Petrotos *et al.* [6] clarified OMWWs by using membrane technology, and the recovered biophenols, after been processed by an reverse osmosis (RO) membrane technique followed by freeze-drying, were encapsulated for the enrichment of yogurt and other dairy products. Troisi *et al.* [7] evaluated the ability of biophenols obtained from OMWWs through ultrafiltration and successive spray drying in controlling the ultra-high-temperature milk treatment processing.

The activity and potential health benefits of the biophenolic compounds are influenced by their stability in end-product formulation and during storage (temperature, oxygen, light) as well as their bioavailability after administration (*i.e.*, insufficient gastric residence time, low permeability, and/or solubility within the gut). The delivery of these compounds therefore requires innovative productive strategies to provide protective mechanisms to preserve their active molecular form and/or to deliver them to target sites in the body. Microencapsulation technology has promoted the development of new products and the improvement of existing products by solving unique challenges, such as converting liquids to solids, separating reactive components, protecting ingredients from the environment, controlling release, or masking ingredients. There have been major advances in the development of delivery systems to encapsulate lipophilic bioactive components, while there is still a pressing need to develop novel manufacturing conditions and formulations to prepare effective delivery systems for hydrophilic bioactives such as biophenols [8].

Major challenges in the design of delivery systems suitable for the encapsulation of bioactive compounds includes the control of particle size and surface properties, as well as the fundamental physicochemical phenomena associated with encapsulation and release (*i.e.*, partitioning and mass transport release of active ingredients) for the production of particle-based end-products with target functionality.

Emulsion systems are essential components of food, cosmetics, and drugs, enhancing the bioavailability of hydrophilic or lipophilic active compounds dissolved in the dispersed water or oil phase, respectively. Monodisperse droplets gives better control over the dose and release behavior of the encapsulated active compounds and yields higher encapsulation efficiency and better biocompatibility. Conventional devices for preparing emulsions such as high pressure, ultrasonic homogenizers, colloid mills, rotor–stator systems, and microfluidizers require high energy input for the production of droplets, leading to droplets with a wide size distribution and a degradation of temperature/shear sensitive compounds that should be encapsulated. Membrane emulsification (ME) is a dispersion process to produce uniform droplets of one liquid phase (e.g., water) in a second immiscible liquid phase (e.g., oil) using a low energy per unit volume [9,10]. The shear stress is applied on the membrane surface, and the droplet size is controlled by the pore size of the membrane [11].

In this work, for the first time, microparticle production, containing biophenols recovered from OMWWs by integrated membrane processes [12], has been achieved. OMWWs used in the present work were produced from the biologic olive oil production (toxic pesticides are not used) and supplied by Olearia San Giorgio (San Giorgio Morgeto, Italy). The process used for the recovery and concentration of biophenols coming from OMWWs has been previously described [12]. Briefly, after suspended solid removal by an acidification/microfiltration (MF) step, concentrated polyphenols where obtained by nanofiltration (NF) followed by osmotic distillation (OD). The enriched biophenolic fraction was used for the preparation of a water-in-oil (W/O) emulsion by ME. Pulsed back-and-forward ME was selected as a low shear encapsulation method because it is particularly attractive for the production of fragile particulate products, such as W/O microemulsions containing shear-sensitive ingredients [13]. Moreover, considering that the bioactive molecule distribution between two phases plays an important role in determining its stability, retention, and release, the coefficient partition of both catechol, as a biomolecule model, and biophenols recovered and concentrated by an advanced membrane process [12] was measured. The feasibility of valorizing biophenols coming from OMWWs in bio-functional particles has here been evaluated. This aspect is a crucial issue with a view to create a drug-controlled delivery system.

## 2. Results

### 2.1. Effect of Dispersed Phase Flux on Particle Size and Size Distribution

**Figure 1:**
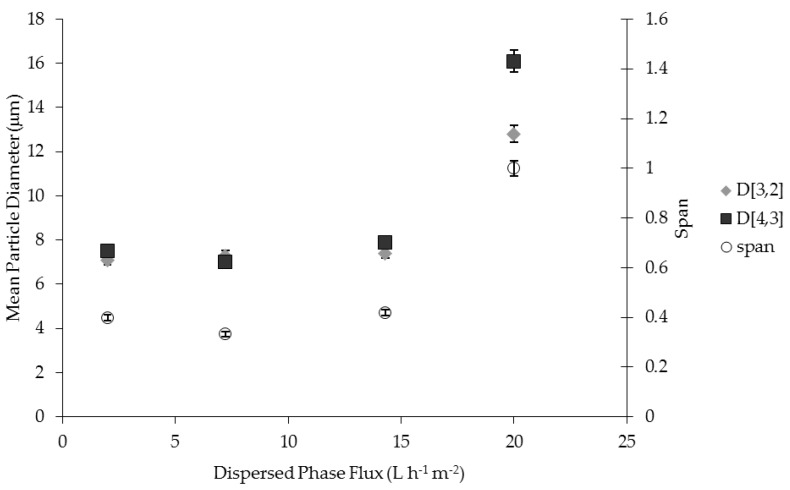
The effect of dispersed phase flux on particle size and particle size distribution of water-in-oil (W/O) emulsion prepared by using pulsed back-and-forward membrane emulsification (shear stress: 5.8 Pa, membrane pore size: 3.1 µm).

The influence of dispersed phase flux on particle size and particle size distribution is shown in Figure 1. Particle size was constant (7.3 µm ± 0.2 µm) in the flux range between 2 to 14.3 L·h^−1^·m^−2^, and uniform drops were produced with a distribution span of 0.4. Particle size (D [3,2] and D [4,3] of 12.8 µm and 16.1 µm, respectively) and particle size distribution (span 1) increases were obtained when the flux was increased to 20 L·h^−1^·m^−2^. Results demonstrated that it is possible to increase the dispersed phase flux up to 14.3 L·h^−1^·m^−2^ without significantly influencing the control of particle production by using the ME process. The shear stress used (5.8 Pa) determined droplet detachment before the increase in droplet volume, giving coalescence at the membrane level, while the further increase in dispersed phase flux leads to the formation of larger droplets before they are detached. In this case, droplet-droplet interactions at the pore level was responsible for broadening the droplet size distribution.

### 2.2. Effect of Shear Stress on Particle Size and Size Distribution

**Figure 2:**
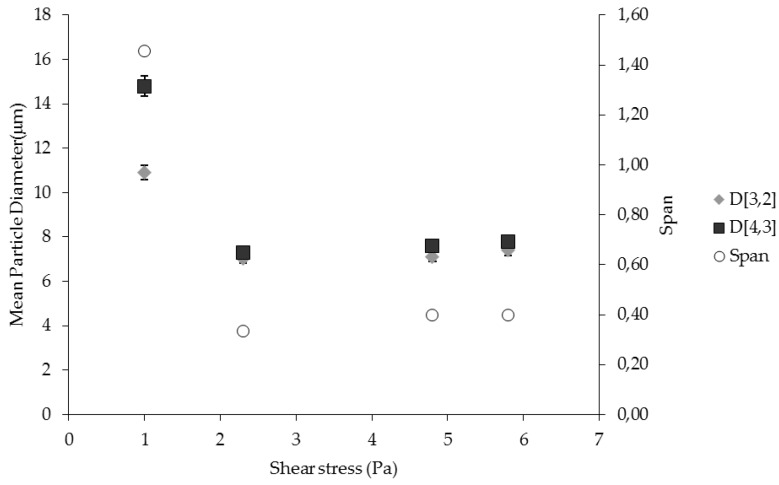
The effect of shear stress on particle size and particle size distribution of W/O emulsion prepared by using pulsed back-and-forward membrane emulsification (dispersed phase flux: 7.2 L·h^−1^·^−2^; membrane pore size: 3.1 µm).

Figure 2 shows the effect of the shear stress on particle size and particle size distribution. It was observed that the largest droplets (D [3,2] = 10.9 µm) were produced at the lowest shear stress value (1 Pa) while in the range between 2.3 and 5.8 Pa the particle size was approximately 2.3 times the membrane pore diameter (7.4 µm). At low shear, larger droplets are produced because they are break away from the membrane not fast enough to prevent the droplet coalescence at the membrane pore level. On the contrary, when the shear stress was increased to 2.3 Pa, droplets detachment is faster and the further increase in the shear does not significantly modify the droplet diameter. Particles with wide size distribution (span = 1.46) were obtained only at low value of shear stress. On the contrary, the particle size distribution was almost independent (span = 0.33–0.40) of the shear in the range between 2.3 to 5.8 Pa because of the decreased probability of droplet coalescence at the membrane surface.

### 2.3. Effect of Dispersed Phase Fraction on Particle Size and Size Distribution

**Figure 3:**
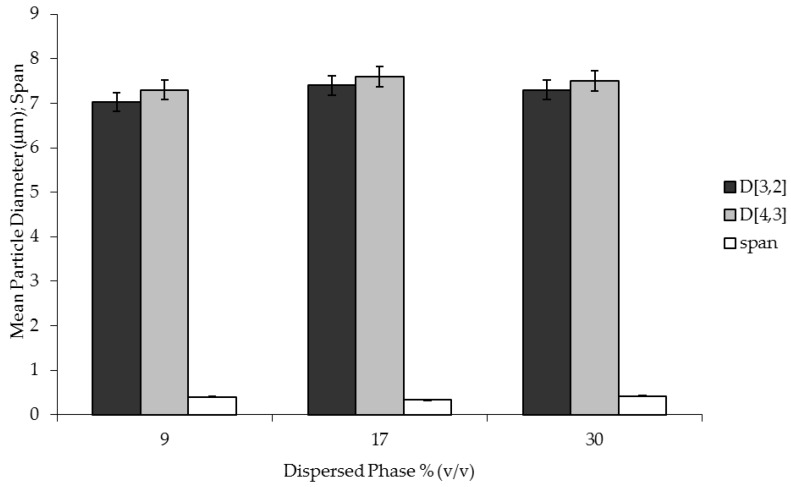
The effect of dispersed phase % (v/v) on particle size and particle size distribution of W/O emulsion prepared by using pulsed back-and-forward membrane emulsification (dispersed phase flux: 7.2 L·h^−1^·m^−2^; shear stress: 5.8 Pa; membrane pore size: 3.1 µm).

The dispersed phase volume fraction in an emulsion plays an important role in determining its stability due to the increased probability of collision frequency or contact between the droplets promoting coalescence, flocculation, or sedimentation. The dispersed phase fraction was changed in the range between 9 to 30% v/v. Figure 3 shows a mean particle diameter and span *versus* the percentage of the water content within the W/O emulsion.

The average size of 7.2 µm for D [3,2] and 7.5 µm for D [4,3] with a span of 0.38 was kept constant when the water content was increased. Because phase separation was not observed, results indicated that the emulsifier (Span 80) accumulates at the interfacial film between the water droplet dispersed phase and the limonene continuous phase, stabilizing the water droplets and avoiding the coalescence mechanism of the water droplet phase. In the emulsification process using membranes, changes in terms of droplet size and size distribution are observed when high concentrations of dispersed phase are reached because of droplet breakup due to the shear during a long operation time [13]. The results demonstrated that it is possible to control the shear stress conditions on the generated drops by appropriately selecting the suitable emulsification membrane-based method to produce highly concentrated formulations with a tuned size and size distribution. The dispersed phase content is important because it determines the initial concentration of active compounds (*i.e.*, flavor molecules, vitamins, drugs) in the emulsion, which has important implications for formulation functional properties (*i.e.*, taste and aroma of food emulsions, and nutraceutical or pharmaceutical activity in an emulsion-based delivery system).

### 2.4. Encapsulation Efficiency and Release Studies

**Figure 4:**
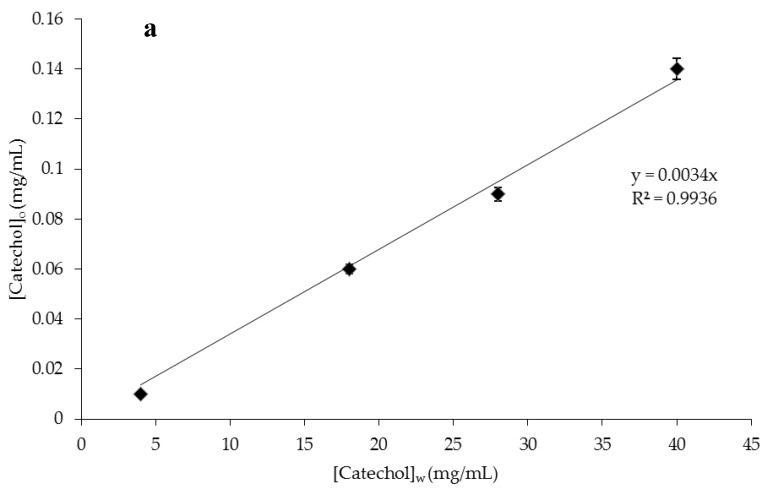

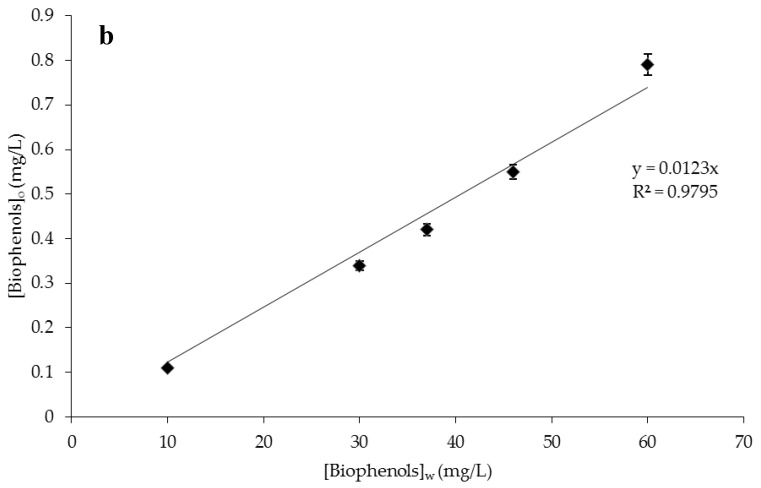
Extraction isotherm at 25 °C between aqueous and limonene phases of (**a**) catechol; (**b**) biophenols coming from olive mill wastewaters (OMWWs).

Catechol and biophenol partition coefficients were determined prior to the release studies. The catechol and biophenols equilibrium isotherms at 25 °C are reported in Figure 4. Figure 4a illustrates a straight line passing from zero, and its slope corresponds to the distribution coefficient of catechol between the two phases, limonene and water. A *K*_D_ of 0.0034 was obtained with an *R*^2^ = 0.9936, showing a good correlation between the data with the fitted regression line. A similar trend was obtained also by calculating polyphenols *K*_D_ using the real OMWWs stream with a *K*_D_ value equal to 0.012 (Figure 4b). Data demonstrate the high affinity of biophenols studied in the present work (catechol and biophenols from OMWWs) toward the water phase.

The different solubility in the two phases and the hydrophilic/lipophilic properties of the biomolecules contained in the real stream are responsible for the higher value of *K*_D_ obtained for biophenols coming from OMWWs. The *K*_D_ of biophenols between the dispersed water phase and the continuous oil phase is a key issue for their successful encapsulation in droplet emulsion and the release that follows. According to this, drug encapsulation efficiency is influenced by the solubility of the drug in the continuous phase. The drug will easily diffuse into the continuous phase or will be retained in the dispersed phase as a function of its solubility in both phases. In emulsion systems, the drug diffusion into the continuous phase occurs until the bioactive partition equilibrium between the two phases is reached. In the present work, the encapsulation efficiency for catechol and biophenols was 98% ± 1% and 92% ± 3%, which corresponds to a partition coefficient between oil and water phases of 0.02 and 0.09, respectively. The partition coefficient estimated in the emulsion system is affected by the compositions of both the continuous phase and the dispersed phase more complex than the limonene/water mixture. However, data confirmed the high affinity of bioactive molecules toward the water phase.

**Figure 5:**
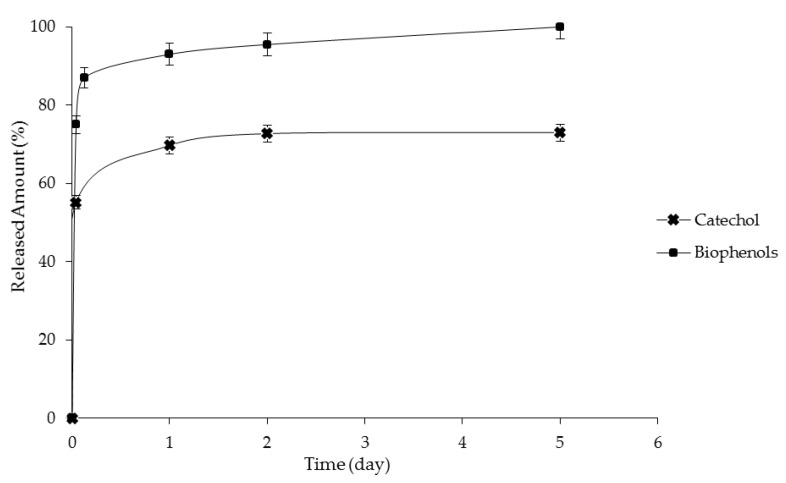
Catechol and biophenols release as a function of time.

The catechol release from the dispersed aqueous phase as a function of time is shown in Figure 5. At the beginning of the release experiment, diffusion of catechol was fast and slowed down slightly after 24 h when the released drug amount of 70% is achieved. A similar trend was observed for the biophenols recovered from OMWWs. Although biophenolic compounds are dissolved in the aqueous dispersed phase previously, they partition among the aqueous and oil components of the emulsion according to the structure and composition of the oil continuous phase (as indicated by the *K*_D_), and they diffuse through the dialysis membrane before being extracted in the receptor solution. As the amount of biophenols from the oil phase is decreased as a consequence of the extraction in the receptor solution, the concentration at equilibrium, corresponding to the partition coefficient, is restored. In these conditions, the emulsion droplets will act as drug reservoirs providing biophenols, which will diffuse across the interfacial film until the equilibrium concentration is restored. The porous cellulose membrane, chosen as dialysis tubing, has a large molecular weight cutoff, which guarantees a minimum barrier effect on the diffusing molecules. The fast release observed in the case of biophenols recovered from OMWWs can be related to the different affinity of each compound of the complex matrix toward the oil phase and the receptor solution.

## 3. Materials and Methods

### 3.1. Materials

A water-in-oil emulsion was prepared using 15 wt % PVA (average MW 13,000–28,000 kDa, Sigma-Aldrich, Milan, Italy) as the dispersed phase and 2 wt % Span 80 (Sigma-Aldrich, Milan, Italy) in limonene (97%, Sigma-Aldrich, Milan, Italy) as the continuous water phase. Catechol (Sigma-Aldrich, Milan, Italy) has been selected as a biophenol molecule model because it was one of the most representative biophenols contained in OMWWs [12] and it was dissolved in a PVA solution. Alternatively, a biophenol-concentrated solution obtained from OMWWs through integrated membrane operations [12] was also used. Considering an initial OMWW volume of 1000 L, it was possible to obtain an enriched fraction of biophenol compounds of 87.5 g/L after taking NF and OD concentration steps. All solvents used for the high-performance liquid chromatography (HPLC) mobile phase preparation (acetonitrile, acetic acid, and methanol) were purchased from Sigma-Aldrich (Milan, Italy).

### 3.2. Preparation of Water-in-Oil Emulsions by Pulsed Back-and-Forward Cross-Flow Batch Membrane Emulsification

**Figure 6:**
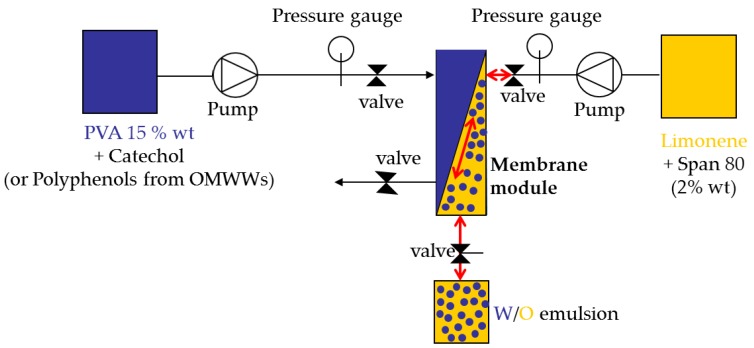
Membrane emulsification plant.

A SPG (Shirasu porous glass) tubular membrane (8.7 mm inner diameter × 0.65 mm wall thickness) with a nominal pore size of 3.1 μm from SPG Technology Co., Ltd. (Miyazaki, Japan) was used. The effective membrane area was 31.3 cm^2^. The membrane was wetted in the continuous phase under vacuum and ultrasonic field before the installation. The schematic figure of the apparatus used for pulsed back-and-forward cross-flow batch membrane emulsification is illustrated in Figure 6.

The dispersed phase was injected from the shell side of the membrane using a peristaltic pump (Gilson, Minipuls 3, 3V Chimica, Rome, Italy). The dispersed phase flux (*J*_d_) was determined by volume upon the water consumption from the graduated feed cylinder. The effect of dispersed phase flux was investigated in the range between 2 to 20 L·h^−1^·m^−2^.

The continuous phase was agitated along the lumen side of the membrane by a programmable peristaltic pump (Digi-Staltic double-Y Masterflex^®^ Micropump, model GJ-N23.JF1SAB1; GENERALCONTROL S.p.A, Milan, Italy) at fixed amplitude and frequency. The maximum shear stress (τ_max_) (Pa) is a function of the amplitude (a) and the frequency (f) of the pulsed flow according to the following equation:

(1) $$\tau_{\text{max}}=2\text{ a }{\left(\pi \text{ f}\right)}^{\frac{3}{2}}\;{\left(\mu_{c}\;\rho_{c}\;\right)}^{\frac{1}{2}}$$

The effect of shear stress was investigated in the range between 1 Pa and 5.8 Pa.

### 3.3. Experimental Setup and Procedure

Preliminary experiments have been carried out in order to identify the appropriate process parameters (dispersed phase flux and shear stress) for membrane emulsification. The operating conditions providing the best uniformity of the droplets have been used for the production of W/O emulsion containing biophenols. Catechol and biophenols (recovered and concentrated from OMWWs) were dissolved in the aqueous dispersed phase. Catechol and biophenol concentration used were 10 g·L^−1^ and 3 g·L^−1^, respectively. The emulsification process was stopped when the dispersed phase percentage was 10% v/v. At the end of the emulsification, 10 mL of emulsion were centrifuged at 10,000 rpm for 10 min to promote phase separation, and the separated water phase was analyzed to measure the concentration of catechol and biophenols (according to the type of encapsulated materials used) via HPLC (Agilent Technologies Italia S.p.A., Milan, Italy) and UV spectrophotometry (Perckin Elmer, Monza, Italy), respectively. The encapsulation efficiency (*EE*%) has been evaluated according to the following equation:

(2) $$EE\%=\frac{C_{0}}{C_{i}}\;\times 100\;$$

where *C*_0_ and *C*_i_ are the measured and initial concentration of catechol (or biophenols) in the aqueous dispersed phase, respectively.

The experimental setup used for release experiments consisted of a cylindrical glass tube put in a thermostatic bath used to keep the temperature at 25 °C. Four milliliters of emulsion containing catechol or biophenols were filled in a dialysis bag (Spectra/Por Dialysis Membrane, *za* < MWCO 15,000) and immersed in 18 mL of a receptor solution (ultrapure water). Aliquots of the receptor solution were withdrawn for the determination of catechol or biophenol concentration with an HPLC and UV spectrophotometer. The aliquot withdrawn was replaced with the same volume of pure water in order to maintain a constant volume of the receptor solution. The amount of catechol or biophenols released (*C*_r_) to the receptor solution is expressed as the ratio between the fraction of catechol (or biophenols) released and their initial encapsulated concentration during the time:

(3) $$C_{r}=\;\frac{C_{t}}{C_{IN}}$$

where *C*_t_ and *C*_IN_ are the concentration of catechol (or biophenols) released at time *t* and initially encapsulated in the aqueous dispersed phase, respectively.

### 3.4. Determination of the Partition Coefficient of Catechol between the Organic Solvent and Water

The bioactive partition between different phases depends on their relative thermodynamic affinity for each phase. Considering that the location of a bioactive within a delivery system plays an important role in determining its stability, retention, and release, the partition (or distribution) coefficient (*K*_D_) of catechol and biophenols was measured.

*K*_D_ gives an indication of the substance solubility in the two phases and is defined by the equilibrium concentration ratio of the component A in the organic ([*A*]*o*) and aqueous phases ([*A*]_w_):

(4) $$K_{D}=\;\frac{{\left[A\right]}_{O}}{{\left[A\right]}_{w}}$$

Another important parameter is the degree of extraction E defined as:

(5) $$E=\;\frac{{\left(n_{A\;}\right)}_{O}}{{\left(n_{A}\right)}_{W}^{IN}}$$

where ${\left(n_{A}\right)}_{W}^{IN}$ = ${\left(n_{A\;}\right)}_{W}$ + ${\left(n_{A\;}\right)}_{O}$ represents the initial moles of the component *A* in the aqueous phase, and ${\left(n_{A\;}\right)}_{O}$ represents the moles at equilibrium.

If *V*_W_ and *V*_O_ are the aqueous and organic phase volumes, then:

(6) $$E=\;\frac{{\left(n_{A\;}\right)}_{O}}{{\left(n_{A\;}\right)}_{W}+\;{\left(n_{A\;}\right)}_{O}}=\;\frac{{\left[A\right]}_{O\;}V_{O}}{{\left[A\right]}_{w}V_{W}+{\left[A\right]}_{O}V_{O}}=\;\frac{\frac{{\left[A\right]}_{O}}{{\left[A\right]}_{w}}}{\frac{{\left[A\right]}_{O}}{{\left[A\right]}_{w}}+\frac{V_{W}}{V_{O}}}$$

The methodology used to calculate the partition coefficients involved the formation of the limonene/water mixtures containing catechol or biophenols. In particular, catechol was dissolved in a known volume of distilled water at concentrations of 5 mg/L and then mixed with limonene; instead, the aqueous solution of biophenols comes from OMWWs. The flasks were immersed in a constant temperature bath (25 °C) and stirred for at least 4 h, long enough to approach equilibrium. After phase separation, catechol concentrations were determined quantitatively in both phases using HPLC. When biophenols from OMWWs were encapsulated, biophenols concentrations were determined by using the Folin-Ciocalteu method.

The distribution coefficient was calculated using different volume ratios of aqueous and organic phases (with a constant initial concentration of catechol in the aqueous phase) [14].

The catechol concentration in limonene was plotted as a function of catechol concentration in the aqueous phase measured at equilibrium after each single extraction. The same procedure was used in the case of biophenols.

### 3.5. Determination of Particle Size and Particle Size Distribution

W/O emulsions were observed with an optical microscope (Zeiss, model Axiovert 25, Carl Zeiss S.p.A., Milan, Italy), equipped with a camera (JVC, model TK-C1481BEG, Carl Zeiss S.p.A., Milan, Italy). Pictures were analyzed by the Scion Image program that allows automatic counting and measurement of the droplets present in a selected area. From these measurements, the mean droplet size and size distribution were evaluated. For each sample, more than 900 droplets were counted and measured.

The mean particle size was expressed as the surface weighted mean diameter (or Sauter diameter), D [3,2], and as the volume weighted mean diameter (or the de Brouckere diameter), D [4,3]. D [3,2] and D [4,3] were determined, respectively, as follows:

(7) $$D\left[3,2\right]=\;\frac{\sum \;D_{i}^{3}\;n_{i}}{\sum \;D_{i}^{2}\;n_{i}}$$

(8) $$D\left[4,3\right]=\;\frac{\sum \;D_{i}^{4}\;n_{i}}{\sum \;D_{i}^{3}\;n_{i}}$$

where *D_i_* = particle diameter of class, and *i* and *n_i_* = number of particle in class *i*. The width of the droplet size distribution was expressed as a Span number, calculated by the following expression:

(9) $$Span=\;\frac{D\left[90\right]-D\left[10\right]}{D\left[50\right]}$$

where *D*[x0] is the diameter corresponding to *x*_0_ vol % on a relative cumulative droplet size curve.

### 3.6. Analytical Measurements

The content of biophenols recovered and concentrated from OMWWs were determined by using the Folin-Ciocalteau method, while HPLC analysis was carried out to evaluate catechol concentration. A HPLC system equipped with an UV detector (Agilent 1200 Series, Agilent Technologies Italia S.p.A., Milan, Italy) and a reversed-phase Luna C18 column (Phenomenex, Torrance, CA, USA) were used. The analysis was carried out at a temperature of 25 °C, a pressure of 100 bar, and a wavelength of 280 nm.

A mixture of water/acetic acid (99/1, v/v) (75%, solvent A) and methanol/acetonitrile/acetic acid (90/9/1, v/v/v) (25%, solvent B) was used as the mobile phase. The analysis was made at a flow rate of 1.0 mL/min.

Total biophenols were estimated colorimetrically by using the Folin-Ciocalteu method. The absorbance was measured using a UV-visible spectrophotometer (Lamda EZ201; Perckin Elmer, Monza, Italy) at 765 nm.

## 4. Conclusions

In the present work, membrane emulsification has been successfully used for the encapsulation of catechol and as a model biophenolic compound, and biophenols have been recovered and concentrated from OMWWs as a real matrix. A W/O emulsion was prepared by using limonene, containing 2 wt % Span 80 as a continuous phase and 15 wt % PVA as a dispersed phase. Results showed that the optimum conditions for preparing uniform emulsion with a controlled droplet size are: a shear stress in the range between 2.3 to 5.8 Pa and a dispersed phase flux in the range between 2 to 14.3 L·h^−1^·m^−2^. Emulsions with a high dispersed phase fraction (30% v/v) were produced without phase separation, and any changes in terms of droplet size and size distribution were observed. High encapsulation efficiency was also obtained for catechol (98% ± 1%) and biophenol fractions purified and concentrated from OMWWs (92% ± 3%), demonstrating that the formulation and the method used in the present work have a potential use for the production of emulsion-based products containing biophenols for applications in the food, pharmaceutical, and cosmetic industries. The presence of additional high-value-added components (such as sugars, fats, and minerals) in the polyphenol-enriched fraction [15,16] as well as the absence of toxic pesticides (due to the use of OMWWs produced from a biologic olive oil production) contribute to an improvement in the quality of encapsulated ingredients for the production of by-products with a potential health benefit.
